# Ethyl 1-benzyl-4-hydr­oxy-2-methyl-5-oxopyrrolidine-3-carboxyl­ate

**DOI:** 10.1107/S1600536810010834

**Published:** 2010-03-27

**Authors:** Graeme J. Gainsford, Jennifer M. Mason

**Affiliations:** a Industrial Research Limited, PO Box 31-310, Lower Hutt, New Zealand

## Abstract

In the title oxopyrrolidine, C_15_H_19_NO_4_, the five-membered pyrrolidine ring is in a twist conformation and its mean plane makes an angle of 89.2 (3)° with the phenyl ring. In the crystal, mol­ecules pack as dimers *via* strong O—H⋯O [*R*_2_^2^(10)] inter­actions cross-linked by weaker C—H⋯O and C—H⋯π inter­actions. Full synthetic and spectroscopic details are given for the title compound and related dicarboxyl­ates.

## Related literature

For details of a programme to elucidate the structure–activity relationships of the Immucillin family of potent purine nucleoside phospho­rylase inhibitors, see: Mason *et al.* (2007 ▶); Edwards *et al.* (2009 ▶); Clinch *et al.* (2009 ▶). For a related structure, see: Snider *et al.* (2000 ▶). For ring conformations see: Cremer & Pople (1975 ▶) and for hydrogen-bond motifs, see: Bernstein *et al.* (1995 ▶).
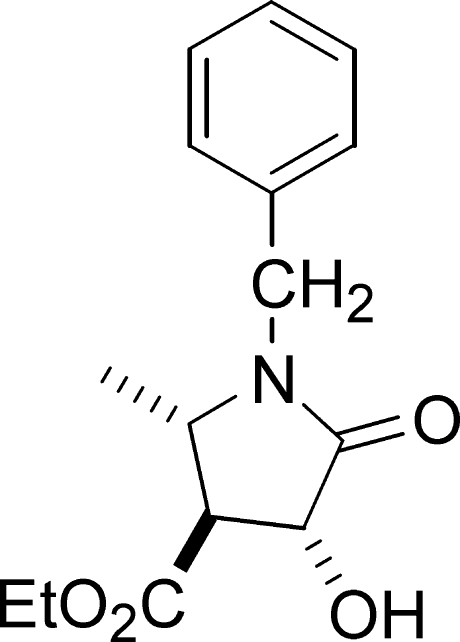


## Experimental

### 

#### Crystal data

C_15_H_19_NO_4_
*M* *_r_* = 277.31
Orthorhombic, 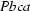

*a* = 27.746 (12) Å
*b* = 14.035 (5) Å
*c* = 7.357 (3) Å
*V* = 2865 (2) Å^3^
*Z* = 8
Mo *K*α radiation
μ = 0.09 mm^−1^
*T* = 93 K
0.45 × 0.14 × 0.01 mm

#### Data collection

Siemens SMART APEX CCD area-detector diffractometer
9428 measured reflections
2376 independent reflections
531 reflections with *I* > 2σ(*I*)
*R*_int_ = 0.136

#### Refinement

*R*[*F* ^2^ > 2σ(*F* ^2^)] = 0.049
*wR*(*F* ^2^) = 0.138
*S* = 1.10
2376 reflections
146 parameters
H atoms treated by a mixture of independent and constrained refinement
Δρ_max_ = 0.26 e Å^−3^
Δρ_min_ = −0.21 e Å^−3^

### 

Data collection: *SMART* (Siemens, 1996 ▶); cell refinement: *SAINT* (Siemens, 1996 ▶); data reduction: *SAINT*; program(s) used to solve structure: *SHELXS97* (Sheldrick, 2008 ▶); program(s) used to refine structure: *SHELXL97* (Sheldrick, 2008 ▶); molecular graphics: *ORTEP* in *WinGX* (Farrugia, 1997 ▶) and *Mercury* (Macrae *et al.*, 2006 ▶); software used to prepare material for publication: *SHELXL97* and *PLATON* (Spek, 2009 ▶).

## Supplementary Material

Crystal structure: contains datablocks global, I. DOI: 10.1107/S1600536810010834/sj2755sup1.cif

Structure factors: contains datablocks I. DOI: 10.1107/S1600536810010834/sj2755Isup2.hkl

Additional supplementary materials:  crystallographic information; 3D view; checkCIF report

## Figures and Tables

**Table 1:** Hydrogen-bond geometry (Å, °) *Cg* is the centroid of the C10–C15 ring.

*D*—H⋯*A*	*D*—H	H⋯*A*	*D*⋯*A*	*D*—H⋯*A*
O2—H2*O*⋯O1^i^	0.93 (5)	1.87 (5)	2.795 (7)	171 (4)
C7—H7*B*⋯O4^ii^	0.99	2.57	3.555 (9)	173
C4—H4⋯O3^iii^	1.0	2.40	3.292 (7)	149
C14—H14⋯*Cg*1^ii^	0.95	2.81	3.612 (8)	142

Symmetry codes: (i) 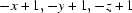; (ii) 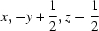; (iii) 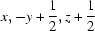.

**Fig. 1.**
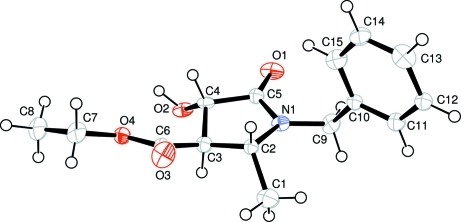
Molecular structure of (I) at the 50% ellipsoid probability level.

**Fig. 2.**
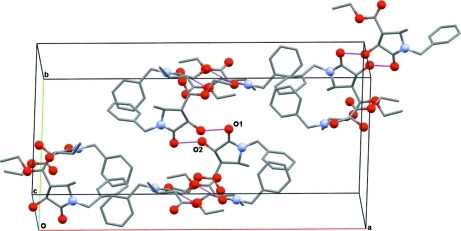
Cell contents for (I) (Mercury, Macrae et al., 2006) showing the strong dimer-forming O–H···O interactions (Table 1).

**Fig. 3.**
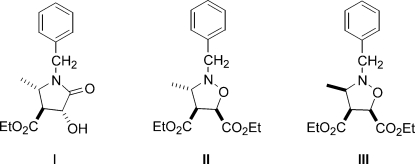
Compounds formed during the synthesis of (I).

## supplementary crystallographic information

### Comment

The title oxopyrrolidine (I) (see Fig 3) was prepared as part of a programme to elucidate the structure-activity relationships of the Immucillin family of potent purine nucleoside phosphorylase inhibitors (Mason *et al.*, 2007; Edwards *et al.*, 2009, Clinch *et al.*, 2009). Cycloaddition of the nitrone formed from *N*-benzyl hydroxylamine and acetaldehyde to diethyl maleate forms racemic, isomeric, isoxazolidine dicarboxylates II and III in 3:1 ratio. Reductive cleavage of the major isomer(II) with zinc was accompanied by spontaneous lactam formation to give the crystalline racemic pyrrolidine (I): the (2*R**,3*R**,4*S**) isomer in shown in Figure 1.

The asymmetric unit of (I), Fig 1, contains one independent ethyl-1-benzyl-4-hydroxy-2-methyl-5-oxopyrrolidine-3-carboxylate(I) molecule. The five-membered ring (*i.e.* N1,C5—C2) is in a twist conformation on C2–C3 with Q(2) 0.260 (7)Å and φ 236.4 (14)° (Cremer & Pople, 1975). Its mean plane makes an angle of 89.2 (3)° with the planar phenyl ring (C10–C15). Distances and angles are similar to those observed before in the related *N*-bis(phenylmethyl)-2-pyrrolydinecarboxamide adduct QECBOP (Snider *et al.*, 2000). Lattice binding is provided principally by O–H···O [motif *R*_2_^2^(10), Bernstein *et al.*, 1995] interactions, shown in Figure 2; these are supported by cross-linking weaker C–H···O and (one) C–H···π interactions (Table 1).

### Experimental

(3*S**,4*S**,5*R**)-Diethyl 2-benzyl-3-methylisoxazolidine-4,5-dicarboxylate (II) and (3*R**,4*S**,5*R**)-Diethyl 2-benzyl-3-methylisoxazolidine-4,5-dicarboxylate (III). Acetaldehyde (0.90 ml, 16.0 mmol) was added to a stirred suspension of *N*-benzyl hydroxylamine (1.8 g, 14.6 mmol) in toluene. After 10 min diethyl maleate ( 2.14 ml, 13.3 mmol) was added and the solution heated to 90°C for 2 h. After cooling the solution was extracted with water, dried and concentrated under reduced pressure. Chromatography of the residue on a column of silica gel eluted with 20 and 25% EtOAc in hexanes gave first (II) (1.6 g, 4.98 mmol, 38%) and then (III) (0.53 g, 1.7 mmol, 12%) as colourless syrups. (II) ESI- MS C_17_H_24_NO_5_ [M+H]^+^ calcd 322.1654, found 322.1638. (III)ESI- MS C_17_H_24_NO_5_ [M+H]^+^ calcd 322.1654, found 322.1666.

(2*S**,3*S**,4*R**)-Ethyl 1-benzyl-4-hydroxy-2-methyl-5-oxopyrrolidine -3-carboxylate(I): Zinc dust (0.52 g, 8.1 mmol) was added to a solution of (3*S**,4*S**,5*R**)-diethyl 2-benzyl-3-methylisoxazolidine-4,5-dicarboxylate (II) (1.3 g, 4.1 mmol) in acetic acid (40 ml). The resulting suspension was stirred overnight and then filtered and concentrated to dryness under reduced pressure. The residue was partitioned between EtOAc and aqueous potassium carbonate (10%). The organic phase was dried and concentrated under reduced pressure. Chromatography of the residue on a column of silica gel eluted with 50-75% EtOAc in hexanes gave the title compound (I) (0.63 g, 56%). Elemental Analysis (%): calcd C 64.97, H 6.91, N 5.05, found C 64.89, H 6.84, N 5.03. Mp (EtOAc-hexanes) 85.9-86.1°C. ESI- MS C_15_H_19_NO_4_Na [M+Na]^+^ calcd 300.1212, found 300.1216.

For full details of ^1^H and ^13^C NMR of compounds (I), (II) & (III) see Special Details in the supplementary data.

### Refinement

The weighting scheme was chosen after the predicted SHELXL parameters gave a significantly poorer distribution of errors over the dataset. The H atom of the ordered hydroxyl group was placed in the position indicated by a difference electron density map and its positions allowed to refine with U_iso_(H) = 1.2U_eq_(O). The methyl H atoms were constrained to an ideal geometry (C—H = 0.98 Å) with U_iso_(H) = 1.5U_eq_(C), but were allowed to rotate freely about the adjacent C—C bonds. All other H atoms were placed in geometrically idealised positions and constrained to ride on their parent atoms with C—H distances of 0.95 (aromatic) or 0.99 (methylene) Å with U_iso_(H) = 1.2U_eq_(C). Two low angle reflections were omitted from the final cycles of refinement because their observed intensities were much lower than the calculated values as a result of being partially obscured by the beam stop. Five other reflections were identified as outliers and removed from refinement.  The crystals were minute in one direction, barely adequate but enough data was measured to solve the structure which met the chemical requirement for the study.

### Crystal data

**Table:**

C_15_H_19_NO_4_	*F*(000) = 1184
*M**_r_* = 277.31	*D*_x_ = 1.286 Mg m^−^^3^
Orthorhombic, *P**b**c**a*	Mo *K*α radiation, λ = 0.71073 Å
Hall symbol: -P 2ac 2ab	Cell parameters from 708 reflections
*a* = 27.746 (12) Å	θ = 2.9–17.8°
*b* = 14.035 (5) Å	µ = 0.09 mm^−^^1^
*c* = 7.357 (3) Å	*T* = 93 K
*V* = 2865 (2) Å^3^	Plate, colourless
*Z* = 8	0.45 × 0.14 × 0.01 mm

### Data collection

**Table:**

Bruker APEXII CCD area-detector diffractometer	531 reflections with *I* > 2σ(*I*)
Radiation source: fine-focus sealed tube	*R*_int_ = 0.136
graphite	θ_max_ = 25.0°, θ_min_ = 2.9°
Detector resolution: 8.333 pixels mm^-1^	*h* = −32→32
φ and ω scans	*k* = −16→16
9428 measured reflections	*l* = −7→7
2376 independent reflections	

### Refinement

**Table:**

Refinement on *F*^2^	Primary atom site location: structure-invariant direct methods
Least-squares matrix: full	Secondary atom site location: difference Fourier map
*R*[*F*^2^ > 2σ(*F*^2^)] = 0.049	Hydrogen site location: inferred from neighbouring sites
*wR*(*F*^2^) = 0.138	H atoms treated by a mixture of independent and constrained refinement
*S* = 1.10	[exp(7.00(sinθ/λ)^2^)]/[σ^2^(*F*_o_^2^) + (0.010*P*)^2^], where *P* = 0.33333*F*_o_^2^ + 0.66667*F*_c_^2^
2376 reflections	(Δ/σ)_max_ < 0.001
146 parameters	Δρ_max_ = 0.26 e Å^−^^3^
0 restraints	Δρ_min_ = −0.21 e Å^−^^3^

### Special details

**Table:**

Experimental. (I)(2*S**,3*S**,4*R**)-Ethyl-1-benzyl-4-hydroxy-2-methyl -5-oxopyrrolidine-3-carboxylate (I) ^1^H NMR (300 MHz, CDCl_3_, TMS) δ 7.35-7.20 (m, 5H), 4.97 (d, *J* = 15.1 Hz, 1H), 4.64 (d, *J* = 8.7 Hz, 1H), 4.18 (q, *J* = 7.1 Hz, 2H), 4.07 (d, *J* = 15.1 Hz, 1H),3.83 (bs, 1H), 3.56 (m, 1H), 2.71 (t, *J* = 8.7 Hz, 1H), 1.33-1.06 (m, 6H). ^13^C NMR (CDCl_3_, 75.5 MHz, centre line of solvent 77.4 ppm) 173.2, 171.6, 136.0, 129.2, 128.3, 128.2, 72.5, 61.9, 54.7, 52.6, 44.6, 19.4, 14.5.(II) (3*S**,4*S**,5*R**)-Diethyl 2-benzyl-3-methylisoxazolidine-4,5-dicarboxylate ^1^H NMR (300 MHz, CDCl_3_, TMS) δ 7.39-7.12 (m, 5H), 4.58 (d, *J* = 8.4 Hz, 1H), 4.17-3.96 (m, 6H), 3.26 (m, 2H), 1.18 (m, 9H). ^13^C NMR (CDCl_3_, 75.5 MHz, centre line of solvent 77.4 ppm) 169.2, 169.0, 137.0, 128.7, 128.1, 127.1, 75.8, 63.8, 60.8, 60.2, 57.4, 16.2, 13.6.(III) (3*R**,4*S**,5*R**)-Diethyl 2-benzyl-3-methylisoxazolidine-4,5-dicarboxylate ^1^H NMR (300 MHz, CDCl_3_, TMS) δ 7.52-7.21 (m, 5H), 4.74 (d, *J* = 9.2 Hz, 1H), 4.32-4.05 (m, 5H), 3.95 (d, *J* = 14.3 Hz, 1H), 3.85 (dd, *J* = 7.6, 9.1 Hz, 1H), 3.30 (m, 1H), 1.86-1.11 (m, 9H). ^13^C NMR (CDCl_3_, 75.5 MHz, centre line of solvent 77.4 ppm) 170.1, 169.5, 136.7, 129.3. 128.6, 127.7, 76.2, 62.6, 61.6, 61.4, 59.6, 55.1, 14.6, 14.4.
Geometry. All esds (except the esd in the dihedral angle between two l.s. planes) are estimated using the full covariance matrix. The cell esds are taken into account individually in the estimation of esds in distances, angles and torsion angles; correlations between esds in cell parameters are only used when they are defined by crystal symmetry. An approximate (isotropic) treatment of cell esds is used for estimating esds involving l.s. planes.
Refinement. Refinement of *F*^2^ against ALL reflections. The weighted *R*-factor wR and goodness of fit *S* are based on *F*^2^, conventional *R*-factors *R* are based on *F*, with *F* set to zero for negative *F*^2^. The threshold expression of *F*^2^ > σ(*F*^2^) is used only for calculating *R*-factors(gt) etc. and is not relevant to the choice of reflections for refinement. *R*-factors based on *F*^2^ are statistically about twice as large as those based on *F*, and *R*- factors based on ALL data will be even larger.

### Fractional atomic coordinates and isotropic or equivalent isotropic displacement parameters (Å^2^)

**Table:**

	*x*	*y*	*z*	*U*_iso_*/*U*_eq_	
O1	0.58333 (18)	0.5432 (2)	0.5104 (6)	0.0312 (13)	
O2	0.4974 (2)	0.5137 (3)	0.2899 (6)	0.0249 (12)	
H2O	0.470 (2)	0.490 (4)	0.347 (7)	0.030*	
O3	0.55079 (18)	0.2657 (3)	−0.0461 (7)	0.0352 (14)	
O4	0.48229 (16)	0.3345 (3)	0.0499 (6)	0.0229 (11)*	
N1	0.6190 (2)	0.4824 (3)	0.2531 (7)	0.0215 (14)	
C1	0.6322 (3)	0.4387 (4)	−0.0757 (8)	0.036 (2)	
H1A	0.6672	0.4340	−0.0602	0.053*	
H1B	0.6237	0.5038	−0.1119	0.053*	
H1C	0.6218	0.3939	−0.1700	0.053*	
C2	0.6070 (2)	0.4141 (4)	0.1053 (9)	0.0195 (16)*	
H2	0.6166	0.3484	0.1436	0.023*	
C3	0.5537 (2)	0.4208 (4)	0.0992 (9)	0.0234 (17)*	
H3	0.5456	0.4744	0.0151	0.028*	
C4	0.5375 (2)	0.4506 (4)	0.2884 (8)	0.0203 (17)	
H4	0.5295	0.3925	0.3612	0.024*	
C5	0.5818 (3)	0.4975 (4)	0.3670 (9)	0.0216 (15)*	
C6	0.5296 (2)	0.3317 (4)	0.0232 (9)	0.0210 (16)*	
C7	0.4550 (2)	0.2497 (4)	−0.0061 (11)	0.0313 (19)	
H7A	0.4625	0.1952	0.0744	0.038*	
H7B	0.4632	0.2319	−0.1326	0.038*	
C8	0.4022 (2)	0.2757 (4)	0.0083 (11)	0.034 (2)	
H8A	0.3950	0.3279	−0.0760	0.051*	
H8B	0.3950	0.2960	0.1329	0.051*	
H8C	0.3825	0.2202	−0.0225	0.051*	
C9	0.6675 (2)	0.5142 (4)	0.2908 (9)	0.0278 (19)	
H9A	0.6664	0.5596	0.3938	0.033*	
H9B	0.6797	0.5493	0.1835	0.033*	
C10	0.7025 (2)	0.4361 (4)	0.3362 (9)	0.0204 (16)*	
C11	0.7490 (3)	0.4379 (4)	0.2743 (9)	0.0260 (16)*	
H11	0.7583	0.4889	0.1969	0.031*	
C12	0.7833 (3)	0.3696 (4)	0.3179 (8)	0.0238 (17)*	
H12	0.8155	0.3733	0.2747	0.029*	
C13	0.7674 (3)	0.2941 (4)	0.4305 (10)	0.033 (2)	
H13	0.7897	0.2458	0.4641	0.039*	
C14	0.7214 (3)	0.2886 (4)	0.4920 (11)	0.031 (2)	
H14	0.7116	0.2362	0.5647	0.037*	
C15	0.6886 (3)	0.3600 (4)	0.4480 (8)	0.0318 (19)	
H15	0.6566	0.3572	0.4941	0.038*	

### Atomic displacement parameters (Å^2^)

**Table:**

	*U*^11^	*U*^22^	*U*^33^	*U*^12^	*U*^13^	*U*^23^
O1	0.057 (4)	0.0099 (18)	0.026 (3)	0.003 (2)	−0.001 (3)	−0.002 (2)
O2	0.042 (3)	0.0100 (19)	0.023 (3)	0.002 (2)	0.006 (3)	0.0030 (19)
O3	0.044 (3)	0.012 (2)	0.050 (3)	0.002 (2)	0.006 (3)	−0.012 (2)
N1	0.033 (4)	0.014 (2)	0.017 (3)	−0.002 (2)	0.002 (3)	−0.006 (2)
C1	0.049 (6)	0.026 (3)	0.032 (4)	−0.005 (4)	−0.003 (5)	0.001 (4)
C4	0.032 (5)	0.014 (3)	0.014 (4)	0.000 (3)	0.002 (4)	0.002 (3)
C7	0.039 (5)	0.010 (2)	0.046 (5)	−0.001 (3)	−0.007 (5)	0.009 (3)
C8	0.041 (5)	0.016 (3)	0.046 (5)	−0.006 (3)	−0.002 (5)	−0.004 (4)
C9	0.035 (5)	0.013 (3)	0.035 (5)	−0.006 (3)	0.001 (4)	−0.004 (3)
C13	0.044 (5)	0.014 (3)	0.039 (5)	0.006 (3)	−0.005 (5)	−0.010 (3)
C14	0.036 (5)	0.021 (3)	0.036 (5)	−0.003 (3)	−0.001 (5)	0.012 (4)
C15	0.047 (5)	0.021 (3)	0.028 (4)	−0.012 (3)	0.000 (5)	−0.008 (3)

### Geometric parameters (Å, °)

**Table:**

O1—C5	1.236 (7)	C7—C8	1.512 (8)
O2—C4	1.421 (7)	C7—H7A	0.9900
O2—H2O	0.94 (6)	C7—H7B	0.9900
O3—C6	1.211 (7)	C8—H8A	0.9800
O4—C6	1.328 (7)	C8—H8B	0.9800
O4—C7	1.471 (6)	C8—H8C	0.9800
N1—C5	1.346 (8)	C9—C10	1.504 (8)
N1—C9	1.442 (8)	C9—H9A	0.9900
N1—C2	1.488 (7)	C9—H9B	0.9900
C1—C2	1.543 (8)	C10—C11	1.367 (9)
C1—H1A	0.9800	C10—C15	1.402 (8)
C1—H1B	0.9800	C11—C12	1.389 (8)
C1—H1C	0.9800	C11—H11	0.9500
C5—C4	1.511 (8)	C12—C13	1.414 (8)
C4—C3	1.522 (8)	C12—H12	0.9500
C4—H4	1.0000	C13—C14	1.357 (9)
C3—C2	1.484 (9)	C13—H13	0.9500
C3—C6	1.523 (8)	C14—C15	1.392 (9)
C3—H3	1.0000	C14—H14	0.9500
C2—H2	1.0000	C15—H15	0.9500
			
C4—O2—H2O	115 (4)	O4—C7—H7A	110.4
C6—O4—C7	116.4 (5)	C8—C7—H7A	110.4
C5—N1—C9	123.1 (6)	O4—C7—H7B	110.4
C5—N1—C2	112.6 (5)	C8—C7—H7B	110.4
C9—N1—C2	123.3 (5)	H7A—C7—H7B	108.6
C2—C1—H1A	109.5	C7—C8—H8A	109.5
C2—C1—H1B	109.5	C7—C8—H8B	109.5
H1A—C1—H1B	109.5	H8A—C8—H8B	109.5
C2—C1—H1C	109.5	C7—C8—H8C	109.5
H1A—C1—H1C	109.5	H8A—C8—H8C	109.5
H1B—C1—H1C	109.5	H8B—C8—H8C	109.5
O1—C5—N1	126.0 (7)	N1—C9—C10	114.8 (5)
O1—C5—C4	125.5 (7)	N1—C9—H9A	108.6
N1—C5—C4	108.6 (5)	C10—C9—H9A	108.6
O2—C4—C5	111.3 (5)	N1—C9—H9B	108.6
O2—C4—C3	114.2 (5)	C10—C9—H9B	108.6
C5—C4—C3	103.2 (6)	H9A—C9—H9B	107.5
O2—C4—H4	109.3	C11—C10—C15	118.0 (6)
C5—C4—H4	109.3	C11—C10—C9	121.5 (6)
C3—C4—H4	109.3	C15—C10—C9	120.5 (7)
C2—C3—C4	106.5 (6)	C10—C11—C12	123.8 (6)
C2—C3—C6	113.4 (5)	C10—C11—H11	118.1
C4—C3—C6	115.6 (6)	C12—C11—H11	118.1
C2—C3—H3	106.9	C11—C12—C13	116.1 (7)
C4—C3—H3	106.9	C11—C12—H12	122.0
C6—C3—H3	106.9	C13—C12—H12	122.0
C3—C2—N1	101.8 (5)	C14—C13—C12	122.0 (7)
C3—C2—C1	114.3 (6)	C14—C13—H13	119.0
N1—C2—C1	112.6 (5)	C12—C13—H13	119.0
C3—C2—H2	109.3	C13—C14—C15	119.8 (7)
N1—C2—H2	109.3	C13—C14—H14	120.1
C1—C2—H2	109.3	C15—C14—H14	120.1
O3—C6—O4	124.4 (6)	C14—C15—C10	120.3 (7)
O3—C6—C3	124.7 (6)	C14—C15—H15	119.9
O4—C6—C3	110.8 (5)	C10—C15—H15	119.9
O4—C7—C8	106.5 (5)		
			
C9—N1—C5—O1	3.8 (10)	C7—O4—C6—O3	1.4 (10)
C2—N1—C5—O1	172.5 (6)	C7—O4—C6—C3	−176.0 (5)
C9—N1—C5—C4	−177.3 (5)	C2—C3—C6—O3	−8.4 (10)
C2—N1—C5—C4	−8.6 (7)	C4—C3—C6—O3	−131.8 (7)
O1—C5—C4—O2	47.3 (8)	C2—C3—C6—O4	169.1 (6)
N1—C5—C4—O2	−131.6 (5)	C4—C3—C6—O4	45.7 (8)
O1—C5—C4—C3	170.2 (6)	C6—O4—C7—C8	−170.9 (6)
N1—C5—C4—C3	−8.7 (6)	C5—N1—C9—C10	108.7 (6)
O2—C4—C3—C2	143.4 (5)	C2—N1—C9—C10	−58.8 (8)
C5—C4—C3—C2	22.5 (6)	N1—C9—C10—C11	140.9 (6)
O2—C4—C3—C6	−89.6 (7)	N1—C9—C10—C15	−40.8 (9)
C5—C4—C3—C6	149.5 (5)	C15—C10—C11—C12	−1.0 (10)
C4—C3—C2—N1	−26.7 (6)	C9—C10—C11—C12	177.4 (6)
C6—C3—C2—N1	−155.0 (5)	C10—C11—C12—C13	1.2 (10)
C4—C3—C2—C1	−148.5 (5)	C11—C12—C13—C14	0.2 (10)
C6—C3—C2—C1	83.3 (7)	C12—C13—C14—C15	−1.7 (11)
C5—N1—C2—C3	22.5 (7)	C13—C14—C15—C10	1.9 (11)
C9—N1—C2—C3	−168.9 (6)	C11—C10—C15—C14	−0.6 (9)
C5—N1—C2—C1	145.4 (5)	C9—C10—C15—C14	−179.0 (6)
C9—N1—C2—C1	−46.0 (8)		

### Hydrogen-bond geometry (Å, °)

**Table:**

Cg is the centroid of the C10–C15 ring.

**Table:**

*D*—H···*A*	*D*—H	H···*A*	*D*···*A*	*D*—H···*A*
O2—H2O···O1^i^	0.93 (5)	1.87 (5)	2.795 (7)	171 (4)
C7—H7B···O4^ii^	0.99	2.57	3.555 (9)	173
C4—H4···O3^iii^	1.0	2.40	3.292 (7)	149
C14—H14···Cg1^ii^	0.95	2.81	3.612 (8)	142

Symmetry codes: (i) −*x*+1, −*y*+1, −*z*+1; (ii) *x*, −*y*+1/2, *z*−1/2; (iii) *x*, −*y*+1/2, *z*+1/2.
